# Necroptosis molecular mechanisms: Recent findings regarding novel necroptosis regulators

**DOI:** 10.1038/s12276-021-00634-7

**Published:** 2021-06-01

**Authors:** Jinho Seo, Young Woo Nam, Seongmi Kim, Doo-Byoung Oh, Jaewhan Song

**Affiliations:** 1 Environmental Disease Research Center, Korea Research Institute of Bioscience and Biotechnology (KRIBB), Daejeon, 34141 Korea; 2 Department of Biochemistry, College of Life Science and Biotechnology, Yonsei University, Seoul, 03722 Korea; 3 Department of Biosystems and Bioengineering, KRIBB School of Biotechnology, University of Science and Technology (UST), Daejeon, 34113 Korea

**Keywords:** Apoptosis, Necroptosis, Glycosylation, Phosphorylation, Ubiquitylation

## Abstract

Necroptosis is a form of programmed necrosis that is mediated by various cytokines and pattern recognition receptors (PRRs). Cells dying by necroptosis show necrotic phenotypes, including swelling and membrane rupture, and release damage-associated molecular patterns (DAMPs), inflammatory cytokines, and chemokines, thereby mediating extreme inflammatory responses. Studies on gene knockout or necroptosis-specific inhibitor treatment in animal models have provided extensive evidence regarding the important roles of necroptosis in inflammatory diseases. The necroptosis signaling pathway is primarily modulated by activation of receptor-interacting protein kinase 3 (RIPK3), which phosphorylates mixed-lineage kinase domain-like protein (MLKL), mediating MLKL oligomerization. In the necroptosis process, these proteins are fine-tuned by posttranslational regulation via phosphorylation, ubiquitination, glycosylation, and protein–protein interactions. Herein, we review recent findings on the molecular regulatory mechanisms of necroptosis.

## Inflammation: Understanding cell death opens therapeutic possibilities

A better understanding of the signaling pathways that drive an inflammatory mode of cell death known as necroptosis could lead to new therapies for autoimmune and neuroinflammatory conditions. Necroptosis is a regulated form of cell death often triggered by infections, tissue injuries, and chronic inflammatory conditions. It leads cells to rupture and disperse their contents, which in turn spurs an inflammatory immune response that can sometimes fuel disease. A team from South Korea led by Jaewhan Song of Yonsei University in Seoul discusses the molecular mechanisms of this process. They focus on several key mediators of the necroptosis signaling pathway and how those proteins in turn are modified to regulate their function. Several drug inhibitors of these proteins are already in clinical development for the treatment of psoriasis and other inflammatory disorders.

## Introduction

Cell death is a terminal physiological event and is intricately related to the maintenance of homeostasis in multicellular organisms. Cell death can be classified into two modes: regulated cell death (RCD) and accidental cell death (ACD). The representative RCD is apoptosis, which plays roles in many physiological processes, such as lymphocyte selection, embryonic development, and epithelium renewal, is modulated by signaling pathways and is identified by cell shrinkage, plasma membrane blebbing, nuclear condensation, and fragmentation of cellular compartments followed by membrane disruption^1^. Apoptosis is initiated by specific intrinsic or extrinsic triggers, including DNA damage, reactive oxygen species, loss of mitochondrial membrane potential, death ligands, and various cytokines, thereby executing cell death in a caspase-dependent manner^1^. In contrast, nonphysiological stimuli, such as physical, mechanical, and chemical stresses, prompt necrosis, which is representative of an ACD^1^. Cells dying by necrosis, which is not regulated by signaling pathways, exhibit swelling, membrane rupture, and secretion of cellular contents^1^. Apoptosis has been regarded as the sole type of RCD since its discovery more than 50 years ago; however, the cell death paradigm has been extended in the past two decades. Although evidence for caspase-independent programmed cell death has been known for a long time, the extensive underlying molecular mechanisms of nonapoptotic cell death have only emerged in the past decade^2^. Since the discovery of necrostatin-1, an inhibitor of receptor-interacting protein kinase 1 (RIPK1) kinase activity, many genetic and chemical inhibition studies have led to the identification of diverse forms of programmed necrosis, including necroptosis, ferroptosis, parthanatos, mitochondrial permeability transition-dependent necrosis, pyroptosis, pyronecrosis, and NETosis^3–5^. In contrast to the passive immune response of cells dying by apoptosis, cells dying by programmed necrosis actively release damage-associated molecular patterns (DAMPs), cytokines, and chemokines, leading to an inflammatory response^5^.

Necroptosis is the best-characterized programmed necrosis. Necroptosis is initiated by various cytokines or pattern recognition receptors (PRRs) and modulated in RIPK1 and RIPK3-dependent manner^6^. RIPK1, RIPK3, and their substrate, mixed-lineage kinase domain-like protein (MLKL), function as key proteins in executing necroptosis, forming a necrosome complex (also referred to as complex II c)^6^. Emerging evidence on the relevance of necroptosis in inflammatory diseases has expanded the roles of necroptosis from those studied in vitro to those studied under pathophysiological conditions^7^. Diverse pathogens, including cytomegalovirus, RNA viruses, and bacteria, are capable of triggering necroptosis by activating Toll-like receptors (TLRs) or Z-DNA-binding protein 1 (ZBP1)^8,9^. In addition to pathogenic infection, necroptosis plays a significant role in tissue injuries such as cardiac, brain, or kidney ischemia-reperfusion injury^4,7,9^. Furthermore, human inflammatory diseases, including atherosclerosis, inflammatory bowel disease, neuroinflammation, and autoimmune diseases, have been closely connected with necroptosis^7^. Since necroptosis plays significant physiological roles, more comprehensive analyses on the interactome effects on necroptosis are required for a thorough understanding of this process.

In the past decade, emerging biochemical and genetic studies, including knockout animal experiments, have illustrated that the necroptosis signaling pathway is fine-tuned by various posttranslational regulations, such as phosphorylation, ubiquitination, glycosylation, and protein–protein interactions. Here, we review the molecular mechanisms of the necroptosis signaling pathway and summarize the recent findings regarding necroptosis regulatory mechanisms.

## Molecular mechanisms of necroptosis

**Fig. 1:**
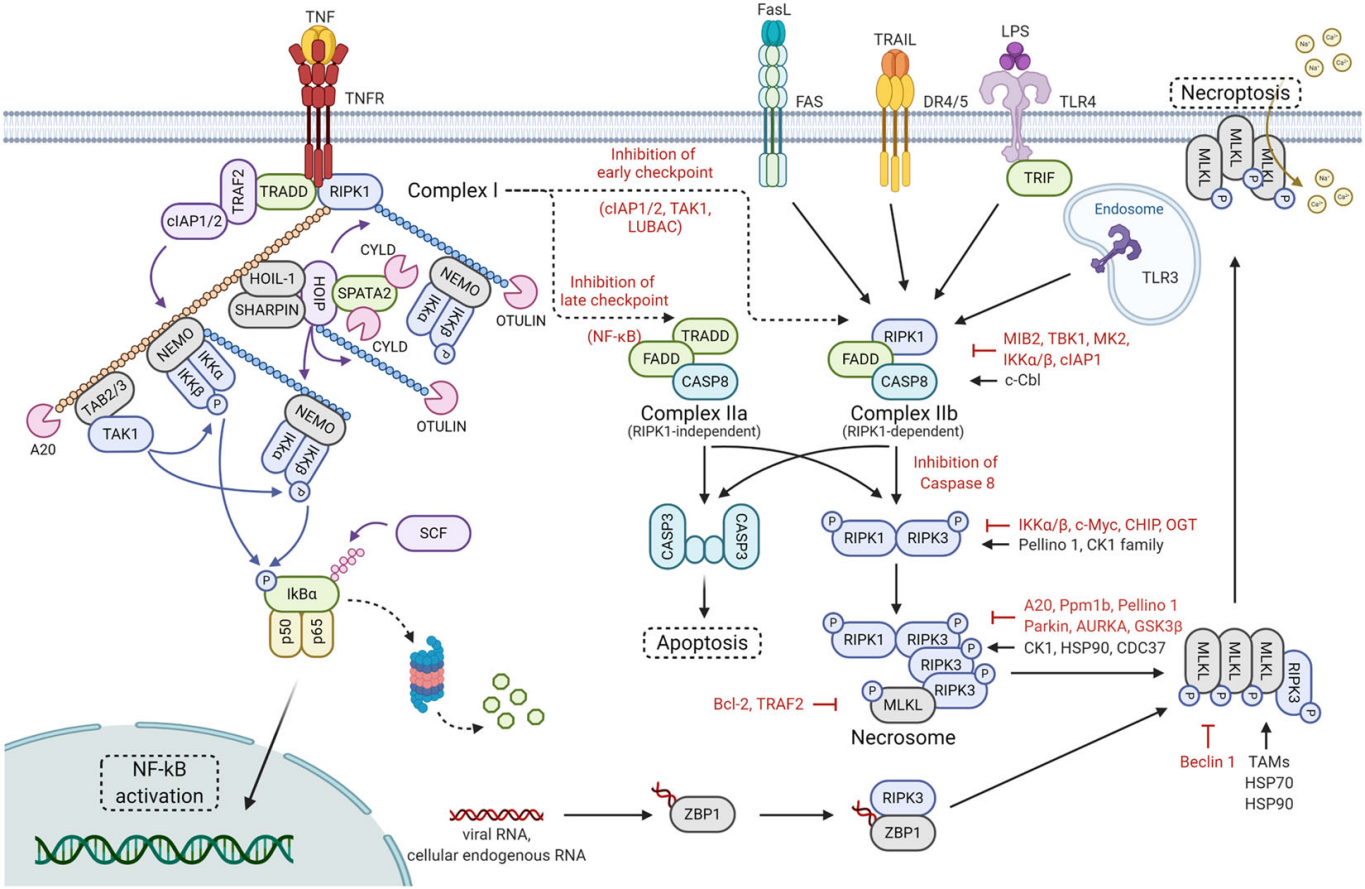
Molecular mechanism of necroptosis. TNF ligation induces complex I formation, which is composed of TRADD, TRAF2, cIAP1/2, RIPK1, TAK1, LUBAC, and the IKK complex, resulting in the activation of the NF-κB signaling pathway. When NF-κB target protein synthesis is inhibited by cycloheximide treatment, complex II a, consisting of TRADD, FADD, and caspase-8, is activated. Caspase-8, activated as part of complex II a, induces apoptosis through the cleavage of downstream molecules. The inhibition of RIPK1 ubiquitination or cytotoxicity-induced inhibition of phosphorylation, the early steps of TNF signaling, results in the induction of complex II b, which is composed of RIPK1, FADD, and caspase-8. Complex II b activation results in the induction of apoptosis through activated caspase-8. When caspase-8 is inhibited, RIPK1 and RIPK3 form necrosome complexes through homotypic interactions with RHIM, resulting in the activation of MLKL through a phosphorylation cascade. Phosphorylated MLKL undergoes oligomerization and migrates to the plasma membrane where it induces necroptosis by initiating membrane rupture or regulating ion flux. Death ligands, including FasL and TRAIL, initiate necroptosis by inducing necrosome complex formation. LPS, poly(I:C), double-stranded RNA, and viral RNA activate necroptosis by TRIF-mediated necrosome complex formation. Viral RNA or cellular endogenous RNA binding to ZBP1 results in RIPK1-independent necroptosis through the ZBP1–RIPK3 complex. Necroptosis factors are strictly regulated by ubiquitination, phosphorylation, glycosylation, and protein–protein interactions.

Most of the studies on the molecular mechanism of necroptosis involve the tumor necrosis factor (TNF) signaling pathway (Fig. 1). Generally, TNF induces an inflammatory response by activating proinflammatory genes through NF-κB signaling. Upon ligation of TNF to its receptor TNF receptor 1 (TNFR1), complex I, consisting of TNFR1-associated death domain protein (TRADD), TNF-receptor-associated factor 2 (TRAF2), RIPK1, cellular inhibitors of apoptosis (cIAP1 or cIAP2), and linear ubiquitin chain assembly complex (LUBAC), is recruited^10^. In complex I, cIAPs and LUBAC promote RIPK1 ubiquitination with K63-linked and linear ubiquitin chains that provide the platforms for recruiting downstream proteins such as TGF-activated kinase 1 (TAK1), TAK1-binding protein 2/3 (TAB2/3), and the IκB kinase (IKK) complex composed of IKKα, IKKβ, and NF-κB essential modulator (NEMO)^10^. The recruited downstream complex activates the NF-κB and mitogen-activated protein kinase (MAPK) pathways, leading to an increase in the expression of proinflammatory genes^10^. However, under certain conditions, such as destabilization of complex I or inhibition of the ubiquitination of RIPK1, TNF can trigger the formation of a cytosolic apoptotic complex (complex II a or II b), including Fas-associated protein with death domain (FADD) and caspase-8, and this complex executes apoptosis^11^.

Disruption of the balance between caspase-8 and RIPK3 activities by caspase-8 inhibition or RIPK3 overexpression promotes conversion of complex II b to necrosome (Fig. 1)^12–14^. RIPK1 and RIPK3 are activated by autophosphorylation or cross-phosphorylation in the necrosome followed by the formation of a large amyloid-like structure^15^. The RIP homotypic interaction motif (RHIM)-dependent RIPK3 oligomer recruits MLKL to the necrosome, subsequently leading to the phosphorylation of MLKL on Thr357 and Ser358 located in the pseudokinase domain^16^. Phosphorylation of MLKL induces a conformational change in MLKL that exposes the 4-helical bundle (4HB) domain, which promotes MLKL oligomerization^17–19^. Oligomerized MLKL moves to the plasma membrane via the Golgi-microtubule-actin machinery, inducing membrane rupture by forming a pore cluster with tight junction proteins or regulating ion channel flux^20–26^.

In addition to TNF, other stimuli can initiate necroptosis (Fig. 1). Death receptors, including Fas (also referred to as CD95 or Apo-1), DR3 (also referred to as Apo-3), DR4 (also referred to as Apo-2 or TRAIL-R1), DR5 (also referred to as TRAIL-R2), and DR6, primarily recruit the membrane-associated death complex consisting of FADD and caspase-8, which is called the death-inducing signaling complex (DISC), upon ligation of the respective ligands^27–30^. In conditions where cIAP and caspase-8 are inhibited, death receptors promote the formation of necrosome, subsequently executing necroptosis in a RIPK3-dependent manner^27–30^. Furthermore, PRRs, such as TLR3 and TLR4, can trigger necroptosis by forming a necrosome through the Toll/IL-1 receptor (TIR) domain-containing adapter protein inducing interferon (IFN)-β (TRIF), which is another RHIM-containing protein^31,32^. In the presence of caspase inhibitors, polyinosine-polycytidylic acid (poly(I:C)) and lipopolysaccharide (LPS) stimulate TLR3 and TLR4 activation, respectively, and active TLRs promote the formation of TRIF-mediated necrosome composed of TRIF, RIPK3, and MLKL, thereby inducing necroptosis^31,32^. Another RHIM-containing protein, ZBP1 (also referred to as DAI), can also initiate necroptosis in response to viral infection^33–35^. Upon sensing viral RNA or cellular endogenous RNA, ZBP1 recruits RIPK3 through an RHIM–RHIM homotypic interaction and then prompts MLKL-dependent and RIPK1-independent necroptosis^33–35^. Recently, the possibility that ZBP1 can initiate necroptosis by the ligation of endogenous dsRNA derived from endogenous retroelements has been suggested^36–38^. RIPK1 deficiency increases ZBP1-mediated necroptosis and inflammation in mice, whereas crossing knock-in mice expressing Zα domain-deleted ZBP1 recovered the inflammatory phenotypes in RIPK1-deficient mice. ZBP1 constitutively binds to endogenous Z-nucleic acid through its Zα domain, suggesting that the Z-nucleic acid sensing of ZBP1 through the Zα domain might be a novel necroptosis initiation mechanism.

### Posttranslational regulation of RIPK1 as a cell fate determinant

**Fig. 2:**
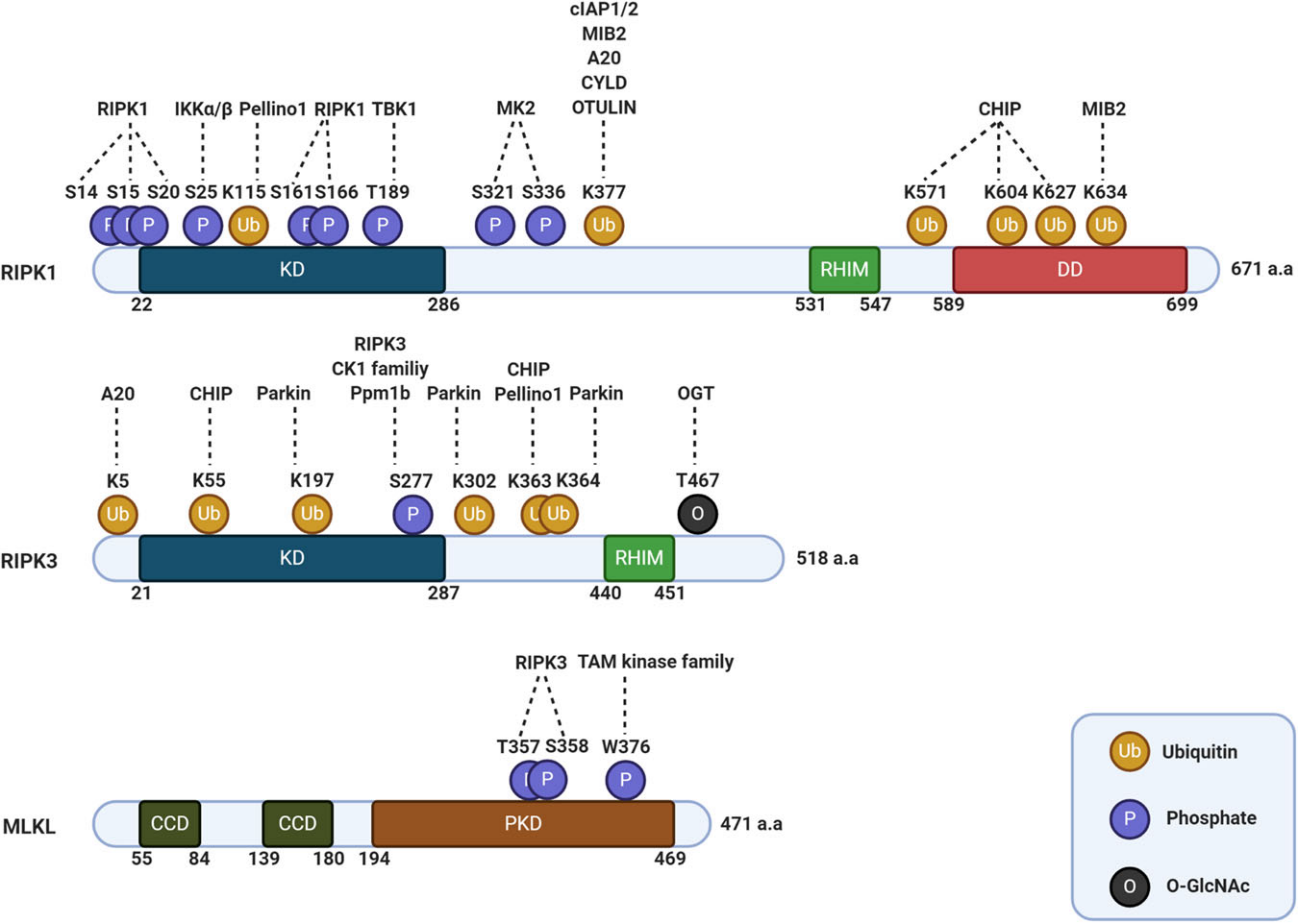
Schematic overview of human RIPK1, RIPK3, and MLKL posttranslational modifications. Overview of RIPK1, RIPK3, and MLKL posttranslational modifications, indicating the amino acid sites for ubiquitination, phosphorylation, and glycosylation with the enzymes have been reported to date.

RIPK1 is a multidomain protein that contains a serine/threonine-protein kinase domain at the N-terminus, an RHIM domain at the intermediate region, and a death domain (DD) at the C-terminus^11^. Following TNF signaling activation, RIPK1 is recruited to complex I via homotypic interactions with other DD-containing proteins^11^. In complex I, RIPK1 is posttranslationally modified by various E3 ligases, deubiquitinases, and kinases, which function as cell fate determinants, regulating the prosurvival, inflammatory, and death signaling pathways (Fig. 2)^10^.

cIAP1 and cIAP2 are RING finger E3 ligases and are recruited to complex I via interaction with TRAF2^6^. cIAP1 and cIAP2 promote K63-linked ubiquitination on RIPK1 Lys377 and their own lysine sites, providing a platform for NF-κB activation (Fig. 2)^39^. The K63-linked ubiquitin chains of cIAPs stabilize complex I. However, when the function of cIAP1 and cIAP2 is inhibited by gene deletion or pharmacological treatment, such as second mitochondria-derived activator of caspase (SMAC) mimetics, the transition of complex I to complex II is promoted, subsequently initiating cell death signaling activation^40^. In addition to K63-linked polyubiquitination of RIPK1, it was recently suggested that cIAP1 is able to regulate the cytotoxic potential of RIPK1 by promoting K48-linked ubiquitination^41^. Cells expressing cIAP1 with a ubiquitin-associated (UBA) domain deletion mutation are more susceptible to TNF-mediated death with a low level of RIPK1 K48-linked ubiquitination and an increase in complex II level^41^. Knock-in mice expressing UBA domain-mutant cIAP1 are more sensitive to TNF-mediated systemic inflammatory response syndrome (SIRS), implying that UBA domain-mediated RIPK1 K48-linked ubiquitination is an important step in modulating the cytotoxic potential of the TNF-mediated response. LUBAC, consisting of heme-oxidized IRP2 ubiquitin ligase 1 (HOIL-1), HOIL-1-interacting protein (HOIP), and shank-associated RH domain-interacting protein (SHARPIN), is a linear ubiquitin E3 ligase complex that engages complex I by interacting with the K63-linked ubiquitin chain of cIAPs in response to TNF activation^42–46^. In complex I, LUBAC conjugates linear ubiquitin chains to RIPK1 and NEMO, which function as docking sites for the IKK complex and another kinase complex composed of NEMO, TANK, NAP1, TBK1, and IKKε, phosphorylating RIPK1 and then restraining the formation of complex II ^42–49^. In contrast to cIAPs and LUBAC, which provide downstream signaling platforms by increasing the ubiquitination of complex I components, MIB2 modulates only the cytotoxic ability of RIPK1 by promoting K11-, K48-, and K63-linked polyubiquitination^50^. Under TNF-activation conditions, MIB2 engages complex I by binding to the linker region of oligomerized RIPK1, which increases RIPK1 ubiquitination on Lys377 and Lys634, thus suppressing the cytotoxic ability of RIPK1 (Fig. 2)^50^. In addition to E3 ligases, counteracting enzymes, deubiquitinases, are regarded as key determinants of cell fate to die or survive. A20, CYLD, and OTU deubiquitinase with linear linkage specificity (OTULIN), which are ubiquitin hydrolases, engage complex I, removing the K63-linked and linear ubiquitination of RIPK1 and other complex I components (Fig. 2)^51–56^. Consistent with their function, deficiency of these enzymes in mice solidifies complex I and the proinflammatory and survival signaling mechanism fails to be terminated, resulting in severe inflammatory phenotypes and eventual death in the early postnatal stage.

In contrast to the cell death-inhibiting ubiquitination described above, ubiquitination of Lys115 of RIPK1 by Pellino 1 promotes necroptosis (Fig. 2)^57^. Pellino 1 conjugates K63-linked polyubiquitination in a RIPK1 kinase activity-dependent manner, which increases the interaction between RIPK1 and RIPK3, subsequently promoting necroptosis^57^. In addition to Pellino 1, c-Cbl can mediate pro-death K63-linked polyubiquitination on RIPK1^58^. When cells are inhibited by a TAK1 inhibitor, complex I recruits leucine-rich repeat serine/threonine-protein kinase 2 (LRRK2), anaphase-promoting complex subunit 11 (APC11), and c-Cbl, increasing K63-linked polyubiquitination of RIPK1. The ubiquitination of RIPK1 induces the formation of a large and insoluble RIPK1 complex (iuRIPK1) and executes RIPK1-dependent apoptosis (RDA) or necroptosis^58^.

Although most RIPK1 ubiquitination is regulated in complex I, cytosolic RIPK1 is also regulated by ubiquitination^59^. Under normal conditions, cytosolic RIPK1 interacts with the carboxy terminus of HSC70-interacting protein (CHIP), which mediates RIPK1 ubiquitination on Lys571, Lys604, and Lys627 (Fig. 2). Ubiquitinated RIPK1 is destabilized in a ubiquitin-lysosome-dependent manner. CHIP deficiency extends the half-life of the RIPK1 protein and increases cell sensitivity to TNF-mediated death, thereby suggesting that CHIP controls steady-state levels of RIPK1^59^.

Although diverse ubiquitin chains on RIPK1 determine its functional roles in TNF-mediated signaling, various kinases are also able to regulate the RIPK1 cytotoxic potential by modulating its kinase activity. IKKα/β, which are recruited to complex I by NEMO, phosphorylate RIPK1 on Ser25, inhibiting RIPK1 kinase-dependent cell death (Fig. 2)^60,61^. Because Ser25 of RIPK1 is located at a Gly-rich loop in the ATP-binding pocket, phosphorylation of RIPK1 at Ser25 seems to inactivate RIPK1 kinase activity by providing electrostatic repulsion at the ATP-binding pocket^61^. Deficiency of either IKKα or IKKβ was insufficient to inhibit RIPK1 phosphorylation or affect RIPK1 kinase-dependent cell death, thereby indicating that these two kinases show functional redundancy^60^. In addition to IKKα/β, TANK-binding kinase 1 (TBK1), also recruited to complex I, can modulate RIPK1 phosphorylation and inhibit its cytotoxic effect (Fig. 2)^62,63^. TBK1/IKKε are recruited to complex I through their interaction with NEMO, NAP1, and TANK, which phosphorylate RIPK1, subsequently suppressing RIPK1 kinase-dependent cell death^62,63^. In contrast to IKKα/β, TBK1/IKKε-mediated RIPK1 phosphorylation does not require TAK1 activation, implying that TBK1/IKKε phosphorylates and modulates RIPK1 in an NF-κB signaling-independent manner^62,63^. Recently, three groups demonstrated that phosphorylation of RIPK1 at Ser321 and Ser336 by MK2 controls RIPK1-dependent cell death (Fig. 2)^64–66^. Under TNF signaling activation, TAK1 phosphorylates p38 and then activates MK2^64–66^. Active MK2 phosphorylates both cytosolic RIPK1 and complex I-recruited RIPK1, restraining the formation of complex II b by blocking its interaction with FADD^64–66^. Although TAK1 indirectly phosphorylates RIPK1 by activating IKKα/β and MK2, direct phosphorylation of RIPK1 by TAK1 has also been demonstrated^67^.

In contrast to the prosurvival effect of RIPK1 phosphorylation mediated by other kinases, the autophosphorylation of RIPK1 causes its enzymatic activation, thus activating its cytotoxic function. Mass spectrometry analysis revealed Ser14/15, Ser20, Ser161, and Ser166 of RIPK1 as autophosphorylation sites (Fig. 2)^68^. Among these, phosphorylation of Ser161 located in the activated T-loop is presumed to induce the conformational change of RIPK1 from the closed form to the open form, leading to enzymatic activation. Consistent with this notion, cells expressing the S161A mutant of RIPK1 showed a slight decrease in kinase activity and RIPK1-dependent necroptosis. Furthermore, reconstitution of the RIPK1 K45A/S161E or D138N/S161E mutant in RIPK1-knockout L929 cells was sufficient to induce RIPK1-dependent necroptosis and necrosome formation, suggesting that autophosphorylation of RIPK1 on Ser161 is an essential step for RIPK1-dependent cell death^69^.

### Posttranslational regulation of RIPK3 in necroptosis modulation

The discovery of the function of RIPK3 in TNF-mediated necrotic cell death was a historic event in cell death studies. For a decade since its discovery, RIPK3 was regarded as merely another RIP-like kinase capable of regulating TNF-mediated apoptosis^70^. However, in 2009, three groups identified RIPK3 as an essential factor in TNF-mediated necroptosis^12–14^. To date, considerable evidence supporting the indispensable role of RIPK3 in necroptosis has emerged.

RIPK3 is an RHIM domain-containing kinase that forms a complex with other RHIM domain-containing proteins, such as RIPK1, ZBP1, and TRIF, under necroptosis signaling activation^6^. In a necrosome, RIPK3 is phosphorylated at multiple sites; among these, autophosphorylation of Ser227 of RIPK3 triggers the recruitment of MLKL to the necrosome by forming a hydrogen bond with Ser404 of MLKL, thereby phosphorylating MLKL and triggering necroptosis (Fig. 2)^16,71^. Recently, two groups have found that members of the casein kinase 1 (CK1) family, serine/threonine kinases, are recruited to the necrosome during necroptosis and phosphorylate RIPK3 on Ser227 (Fig. 2)^72,73^. Loss of CK1 blocked necroptosis with a decrease in RIPK3 phosphorylation at Ser227, suggesting that CK1 family proteins may play important roles in the promotion of necroptosis^73^. Along with kinases and their counterenzymes, phosphatases have been identified as regulators of necroptosis. Using mass spectrometry, Ppm1b was isolated as a binding partner of RIPK3 that dephosphorylates RIPK3 (Fig. 2)^74^. Consistent with this finding, Ppm1b-deficient mice showed increased lethality in TNF-induced SIRS with augmented RIPK3 phosphorylation, implying that RIPK3 dephosphorylation by Ppm1b functions as a negative regulator under physiological conditions.

Ubiquitination of RIPK3 is required to stabilize the necrosome complex. Lys5 of RIPK3 was identified as the ubiquitination site by mass spectrometry analysis (Fig. 2)^75^. Cells expressing the K5A mutant of RIPK3 showed neither RIPK3 ubiquitination nor necroptotic cell death. Although the E3 ligase of RIPK3 remains unknown, the ubiquitination of RIPK3 on Lys5 was negatively regulated by A20. Furthermore, the necroptotic phenotypes of A20-deficient T cells were restored by the combination knockout that included abrogation of RIPK3, indicating that the ubiquitination of RIPK3 in the necrosome might be an important checkpoint in executing necroptosis.

Cytosolic RIPK3 is also regulated by ubiquitination. CHIP, which is also known as a RIPK1 E3 ligase, interacts with RIPK3, subsequently ubiquitinating Lys55 and Lys363 of RIPK3 (Fig. 2)^59^. Ubiquitinated RIPK3 is broken down by lysosome-mediated degradation. CHIP-deficient newborn mice die in 4 weeks with disruption of the intestine, whereas CHIP-deficient mice with RIPK3 knocked out overcome postnatal lethality with normal intestinal physiology. In addition to CHIP, Pellino 1 can regulate RIPK3 steady-state levels by increasing ubiquitination^76^. Thr182 phosphorylation of RIPK3, which is mediated by necroptotic stimuli, is required for the interaction between Pellino 1 and RIPK3, which is followed by RIPK3 ubiquitination. In contrast to CHIP-mediated ubiquitination, Pellino 1-mediated RIPK3 ubiquitination leads to protein degradation in a proteasome-dependent manner. Parkin was also identified as a RIPK3 E3 ligase^77^. During the necroptosis process, AMP-activated protein kinase (AMPK) is activated by RIPKs, subsequently phosphorylating Ser9 of Parkin. Phosphorylated Parkin causes K33-linked polyubiquitination on Lys197, Lys302, and Lys364 of RIPK3, thereby inhibiting necrosome formation (Fig. 2). Loss of Parkin in mice led to an increased incidence of colitis-associated cancer with elevated inflammation induced by DSS, and treatment with GSK′872, a RIPK3 inhibitor, reversed the effect of Parkin deficiency.

Recently, the *O*-Glc*N*Acylation of RIPK3 on Thr467 was identified as a posttranslational modification (PTM) inhibiting the homotypic interaction of the RHIM domain (Fig. 2)^78^. Under LPS treatment conditions, the interaction between *O*-Glc*N*Ac transferase (OGT) and RIPK3 increased in a time-dependent manner with an increase in the *O*-Glc*N*Acylation of RIPK3, whereas the interaction between the two proteins was diminished with a decrease in *O*-Glc*N*Acylation of RIPK3 when cells were treated with LPS and z-VAD-FMK. Glc*N*Ac on Thr467 is presumed to block the homotypic interaction of the RHIM domain by inducing steric hindrance. Consistent with this supposition, the inflammatory response of macrophage-specific OGT-deficient mice in response to septic shock was recovered by RIPK3 codeletion.

In addition to PTMs, several proteins can inhibit necroptosis by interfering with the RIPK1–RIPK3 interaction. Aurora kinase A (AURKA) and GSK3β interact with RIPK1 and RIPK3, thereby negatively regulating necroptosis by interfering with necrosome formation^79^. The heat-shock protein 90 (HSP90)–CDC37 complex is also able to regulate RIPK3 by protein–protein interactions and is required for the interaction between RIPK1 and RIPK3 in response to necroptotic stimuli^80^. HSP90 inhibitor treatment alleviates TNFα-induced tissue damage in vivo. In addition to HSP90–CDC37, MYC, which is an oncogene, can negatively regulate necroptosis by blocking the interaction between RIPK1 and RIPK3^81^. MYC directly interacts with RIPK3 under normal conditions and interferes with necrosome formation. Loss of MYC sensitizes cells to necroptosis with an increase in RIPK1 and RIPK3 interaction, and reconstitution of MYC in MYC-knockout cells restores cell sensitivity to necroptotic stimuli such that it is similar to that of normal cells. Interestingly, the expression of MYC mutants with deficient nuclear function in MYC-knockout cells can also reestablish the cytotoxic effect of MYC, implying that MYC negatively regulates necroptosis in a transcriptional function-independent manner. Consistent with this notion, xenograft analysis showed that MYC deficiency increased leukemia cells sensitivity to SMAC mimetic and pan-caspase inhibitor treatment, thus suggesting that the combination treatment of a MYC inhibitor with a SMAC mimetic and a pan-caspase inhibitor might be a good therapeutic strategy for leukemia patients.

### Posttranslational regulatory mechanism of MLKL

In 2012, a mass spectrometry analysis revealed that MLKL and phosphoglycerate mutase family 5 (PGAM5) were downstream molecules of RIPK3^16,82^. Although the necroptotic function of PGAM5 has been challenged by gene-deletion studies, MLKL is an unquestionable downstream executor of necroptosis. MLKL contains 4HB in its N-terminus and a pseudokinase domain at its C-terminus. Under RIPK3 activation, MLKL is recruited to the necrosome through its interaction with RIPK3 and is subsequently phosphorylated at the Thr357 and Ser358 in the pseudokinase domain (Fig. 2)^16^. This MLKL phosphorylation by RIPK3 leads to a conformational change in which the 4HB domain changes from the masked form to the exposed form, causing MLKL oligomerization^17,19–24,71,83^. Recently, TYRO3, AXL, and MER receptor kinases (TAMs) were identified as MLKL kinases^84^. TAMs phosphorylate Tyr376 of MLKL under necroptotic stress, which promotes MLKL oligomerization (Fig. 2). Notably, loss of TAMs attenuated necroptosis without changing the phosphorylation of RIPK1, RIPK3, or Ser358 in MLKL, implying that the phosphorylation of MLKL by TAMs might be the final modification step in the necroptosis process.

In addition to kinases, several proteins can regulate MLKL function through protein–protein interactions. TRAF2, an adapter protein in complex I, constitutively interacts with MLKL under normal conditions and suppresses the recruitment of MLKL to the necrosome in response to necroptotic stimuli^85^. While TRAF2-inducible knockout mice died within a week of induction because of an increase in necrosome formation, RIPK3 codeletion delayed the lethality of the TRAF2-inducible knockout mice. HSP90, a molecular chaperone, has also been identified as a regulator of MLKL^86,87^. The inhibition of HSP90 by specific inhibitors blocks necroptosis with a decrease in MLKL oligomerization and protein stability, suggesting that HSP90 may be required for the maintenance of MLKL oligomerization and protein stability. In addition HSP90, HSP70 has recently been found to be a positive regulator of MLKL^88^. The substrate-binding domain (SBD) of HSP70 is able to bind to the N-terminal domain (NTD) of MLKL, promoting MLKL polymerization and stabilization. The inhibition of HSP70 by treatment with NBC1, which can bind to the SBD of HSP70, prevents MLKL polymerization without inhibiting MLKL tetramerization, suggesting that HSP70 regulates necroptosis by modulating the MLKL polymerization step after MLKL tetramerization.

MLKL contains protein-binding regions such as the BH3-like motif and the coiled-coil domain (CCD) in its N-terminal region. Bcl-2, which is a BH3 motif-containing antiapoptotic protein, was identified as a negative regulator of MLKL^89^. Bcl-2 can bind to the BH3-like motif located at Lys165 to Lys177 of MLKL, interfering with RIPK3-mediated MLKL phosphorylation and MLKL oligomerization. Recently, the autophagic core protein Beclin 1 was identified as an inhibitory member of the necrosome^90^. Beclin 1 engages the necrosome during necroptosis by interacting with MLKL. The CCD is critical for the interaction of Beclin 1 with MLKL, inhibiting necroptosis by suppressing MLKL oligomerization. The necroptosis-inhibiting function of Beclin 1 proceeds in an autophagy-independent manner. MLKL phosphorylation by RIPK3 is required for the Beclin 1 and MLKL interaction, implying that Beclin 1 functions as the final barrier to the necroptosis process. In addition to in vitro analysis, tests on the inhibitory function of Beclin 1 in vivo were performed using a leukemia cell-based xenograft animal model. Beclin 1-defective leukemia cells were more sensitive to necroptotic stimuli in vitro and in vivo with increased MLKL oligomerization, suggesting that Beclin 1 might be a good target for the treatment of leukemia patients.

## Concluding remarks

Currently, necroptosis is recognized as a representative RCD, as is apoptosis. In the past decade, the relevance of necroptosis to inflammatory diseases has been proven using animal models, including gene knockout mice and necroptosis-inhibiting treatment^7^. The inhibition of necroptosis by gene knockout or pharmacological treatment alleviated SIRS, ischemic-reperfusion injury, and other inflammatory responses^7^. Diverse necroptosis inhibitors have been discovered because of the increasing recognition of their importance under physiological conditions. Necrostatin-1 and its derivative were identified as the first necroptosis inhibitors that block RIPK1 kinase activity^68,91^. After the discovery of necrostatin-1, GlaxoSmithKline (GSK) found highly specific necroptosis inhibitors, namely, GSK′963 (a RIPK1 kinase inhibitor), GSK′843, and GSK′872 (RIPK3 kinase inhibitors)^92,93^. Moreover, necrosulfonamide has been reported as an MLKL inhibitor^16^. In addition to these newly generated chemicals, many approved medicines have been found to be necroptosis inhibitors^94^. Most of the approved medicines seem to target RIPK1 or RIPK3, indicating the importance of RIPKs in necroptosis. Currently, several RIPK1 inhibitors, GSK′2982772, DNL747, and DNL758, are in phase 1 or 2 clinical trials targeting inflammatory diseases such as psoriasis, ulcerative colitis, severe rheumatoid arthritis, Alzheimer’s disease, amyotrophic lateral sclerosis, and COVID-19^95,96^.

As diverse diseases continue to be connected with necroptosis, the significance of understanding necroptosis regulatory mechanisms is increasing (Table 1). Although various factors have been revealed as posttranslational regulatory molecules since the discovery of necroptosis, several posttranslational modifications, such as the ubiquitination of RIPK3 and MLKL in the necrosome, remain unclear. Furthermore, cross talk between other signaling molecules and necroptosis has recently been reported, suggesting that diverse cellular signaling pathways might be intricately entangled with the necroptosis signaling pathway. Thus, elucidation of these regulatory networks in necroptosis is required to obtain a comprehensive understanding of the pathophysiological roles of necroptosis in inflammatory diseases, which will ultimately result in the development of novel therapeutic strategies for the management of inflammatory diseases.

**Table 1:** Posttranslational regulation of necroptosis by ubiquitination, phosphorylation, glycosylation, and protein–protein interactions.

Target	Protein	Site	Death	Function	Ref.
RIPK1	cIAP1, cIAP2	K377	↓	cIAP1 and cIAP2 mediate K63-linked ubiquitination on Lys377 of RIPK1, providing a platform of the NF-κB signaling pathway. In addition, cIAP1 mediates K48-linked ubiquitination on of RIPK1 and decreases complex II formation.	^39,41^
	LUBAC	N/D	↓	LUBAC mediates linear ubiquitination of RIPK1, activates NF-κB signaling, and restrains complex II formation.	^42–49^
	MIB2	K377, 634	↓	MIB2 mediates K11-, K48-, K63-linked ubiquitination on Lys377 and Lys634 of RIP1, and suppresses the cytotoxic ability of RIPK1.	^50^
	A20, CYLD, OTULIN	K377	↑	A20, CYLD, and OTULIN deubiquitinates K63 and M1-linked ubiquitin chain on Lys377 of RIPK1, and turn off the NF-κB signaling pathway.	^51–56^
	Pellino 1	K115	↑	Pellino 1 mediates K63-linked ubiquitination on of RIPK1 and increases the interaction between RIPK1 and RIPK3.	^57^
	c-Cbl	N/D	↑	c-Cbl mediates K63-linked ubiquitination on of RIPK1 and induces iuRIPK1 formation.	^58^
	CHIP	K571, 604, 627	↓	CHIP ubiquitinates Lys571, Lys604, and Lys672 on RIPK1, and induces lysosomal degradation of RIPK1.	^59^
	IKKα/β	S25	↓	IKKα/β phosphorylate Ser25 on RIPK1 and inactivate kinase activity of RIPK1.	^60,61^
	TBK1	T189	↓	TBK1 phosphorylates Thr189 on RIPK1 and suppresses kinase activity of RIPK1.	^62,63^
	MK2	S321, 336	↓	MK2 phosphorylates Ser321 and Ser336 on RIPK1 and restrains formation of complexx II b.	^64–66^
	RIPK1	S14, 15, 20, 161, 166	↑	RIPK1 autophosphorylates Ser14, Ser15, Ser20, Ser161, and Ser166 and leads to enzymatic activation of RIPK1.	^68^
RIPK3	A20	K5	↓	A20 deubiquitinates Lys5 on RIPK3 and suppresses necrosome formation.	^75^
	CHIP	K55, 363	↓	CHIP deubiquitinates Lys55 and Lys363 on RIPK3 and induces lysosomal degradation of RIPK3	^59^
	Pellino 1	K363	↓	Pellino 1 recognizes Thr182 on phosphorylated-RIPK3, mediates K48-linked ubiquitination, and induces proteasomal degradation of RIPK3.	^76^
	Parkin	K197, 302, 364	↓	Parkin mediates K33-linked ubiquitination on Lys197, Lys302, and Lys364 of RIPK3, and suppresses necrosome formation.	^77^
	RIPK3	S227	↑	RIPK3 phosphorylates Ser277 on RIPK3 and activates kinase activity of RIPK3	^71^
	CK1	S227	↑	CK1 family phosphorylate Ser277 on RIPK3 and activates kinase activity of RIPK3.	^72,73^
	Ppm1b	S227	↓	Ppm1b dephosphorylates Ser227 on RIPK3 and decreases RIPK3 activation.	^74^
	OGT	T467	↓	OGT mediates *O*-Glc*N*Acylation on Thr467 of RIPK3 and induces dysfunction of its RHIM domain.	^78^
	AURKA, GSK3β	N/D	↓	AURKA, GSK3β are recruited in necrosome complex and suppress the interaction between RIPK1 and RIPK3.	^79^
	HSP90-CDC37	N/D	↑	HSP90-CDC37 complex is required for the necrosome complex formation.	^80^
	MYC	Kinase, RHIM	↓	MYC binds to RIPK3 and interprets interaction between RIPK1 and RIPK3	^81^
MLKL	RIPK3	T357, S358	↑	RIPK3 induces a conformational change of MLKL and oligomerization of MLKL.	^16^
	TAM	W376	↑	TAM kinase family phosphorylate Trp376 on MLKL and increases oligomerization of MLKL	^84^
	Bcl-2	165-176	↓	Bcl-2 binds to MLKL and interferes with the interaction between RIPK3 and MLKL.	^89^
	TRAF2	N/D	↓	TRAF2 binds to MLKL and suppresses phosphorylation of MLKL by RIPK3.	^85^
	HSP70	N/D	↑	HSP70 increases oligomerization of MLKL and maintains MLKL protein stability.	^88^
	HSP90	N/D	↑	HSP90 modulates MLKL polymerization via substrate-binding domain.	^86,87^
	Beclin 1	4HB	↓	Beclin 1 is recruited to necrosome and suppresses oligomerization of MLKL.	^90^

*N/D* not determined.
